# HIV-1 Impact on Malaria Transmission: A Complex and Relevant Global Health Concern

**DOI:** 10.3389/fcimb.2021.656938

**Published:** 2021-04-12

**Authors:** Ashleigh Roberds, Emily Ferraro, Shirley Luckhart, V. Ann Stewart

**Affiliations:** ^1^ Department of Preventive Medicine and Biostatistics, Division of Tropical Public Health, Uniformed Services University of the Health Sciences, Bethesda, MD, United States; ^2^ Department of Preventive Medicine and Biostatistics, F. Edward Hébert School of Medicine, Uniformed Services University of the Health Sciences, Bethesda, MD, United States; ^3^ Department of Entomology, Plant Pathology and Nematology, Department of Biological Sciences, College of Agricultural and Life Sciences, University of Idaho, Moscow, ID, United States

**Keywords:** malaria, transmission, HIV-1, *Plasmodium falciparum*, co-infection, gametocytes, HIV, review (article)

## Abstract

Malaria/HIV-1 co-infection has become a significant public health problem in the tropics where there is geographical overlap of the two diseases. It is well described that co-infection impacts clinical progression of both diseases; however, less is known about the impact of co-infection on disease transmission. Malaria transmission is dependent upon multiple critical factors, one of which is the presence and viability of the sexual-stage gametocyte. In this review, we summarize evidence surrounding gametocyte production in *Plasmodium falciparum* and the development factors and the consequential impact that HIV-1 has on malaria parasite transmission. Epidemiological and clinical evidence surrounding anemia, immune dysregulation, and chemotherapy as it pertains to co-infection and gametocyte transmission are reviewed. We discuss significant gaps in understanding that are often due to the biological complexities of both diseases as well as the lack of entomological data necessary to define transmission success. In particular, we highlight special epidemiological populations, such as co-infected asymptomatic gametocyte carriers, and the unique role these populations have in a future focused on malaria elimination and eradication.

## Introduction

**Figure 1:**
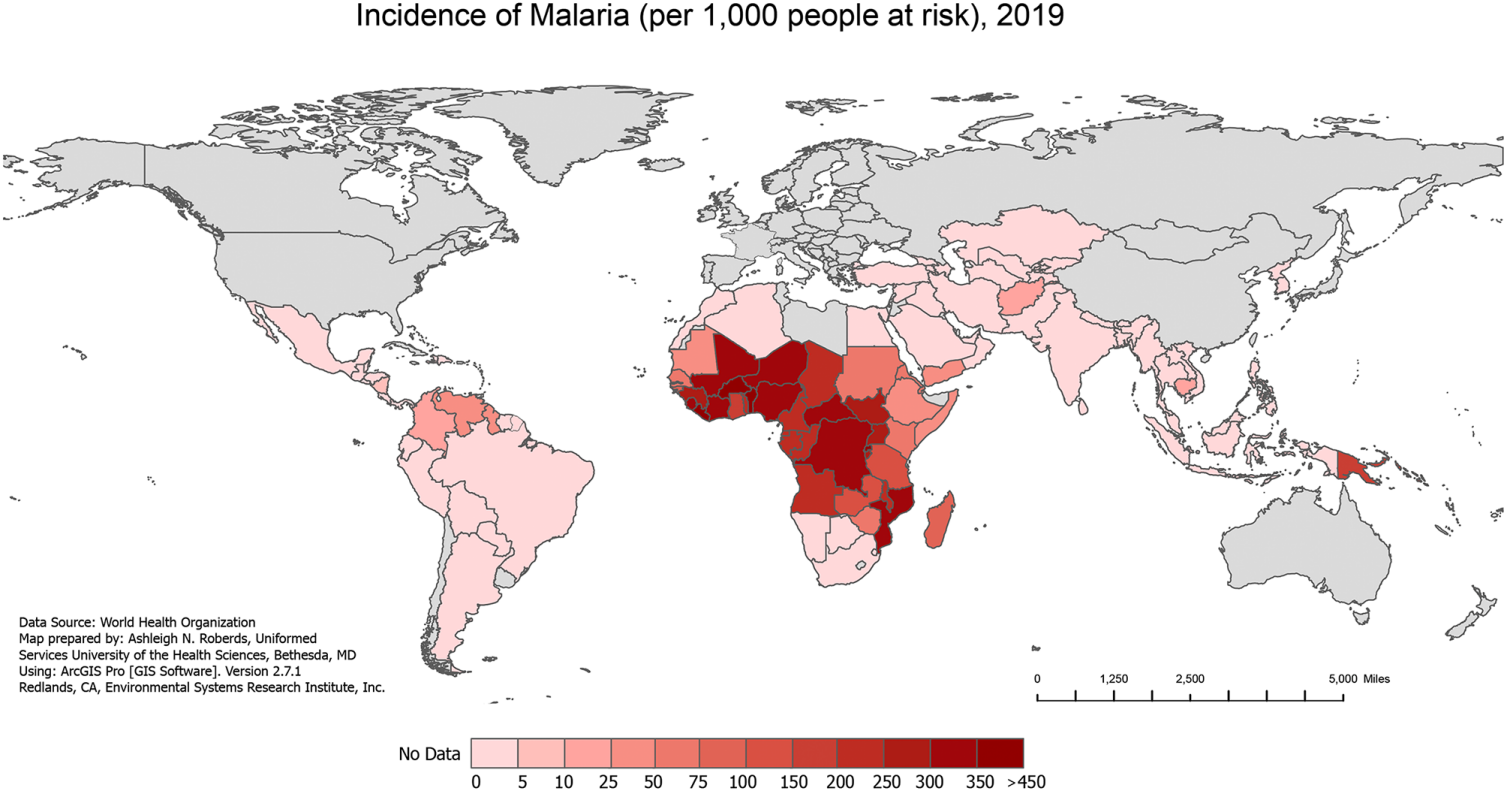
Incidence of Malaria, 2019. The incidence of all malaria cases per 1,000 people at risk in 2019, extracted from the World Health Organization (WHO) 2020 World Malaria Report.

**Figure 2:**
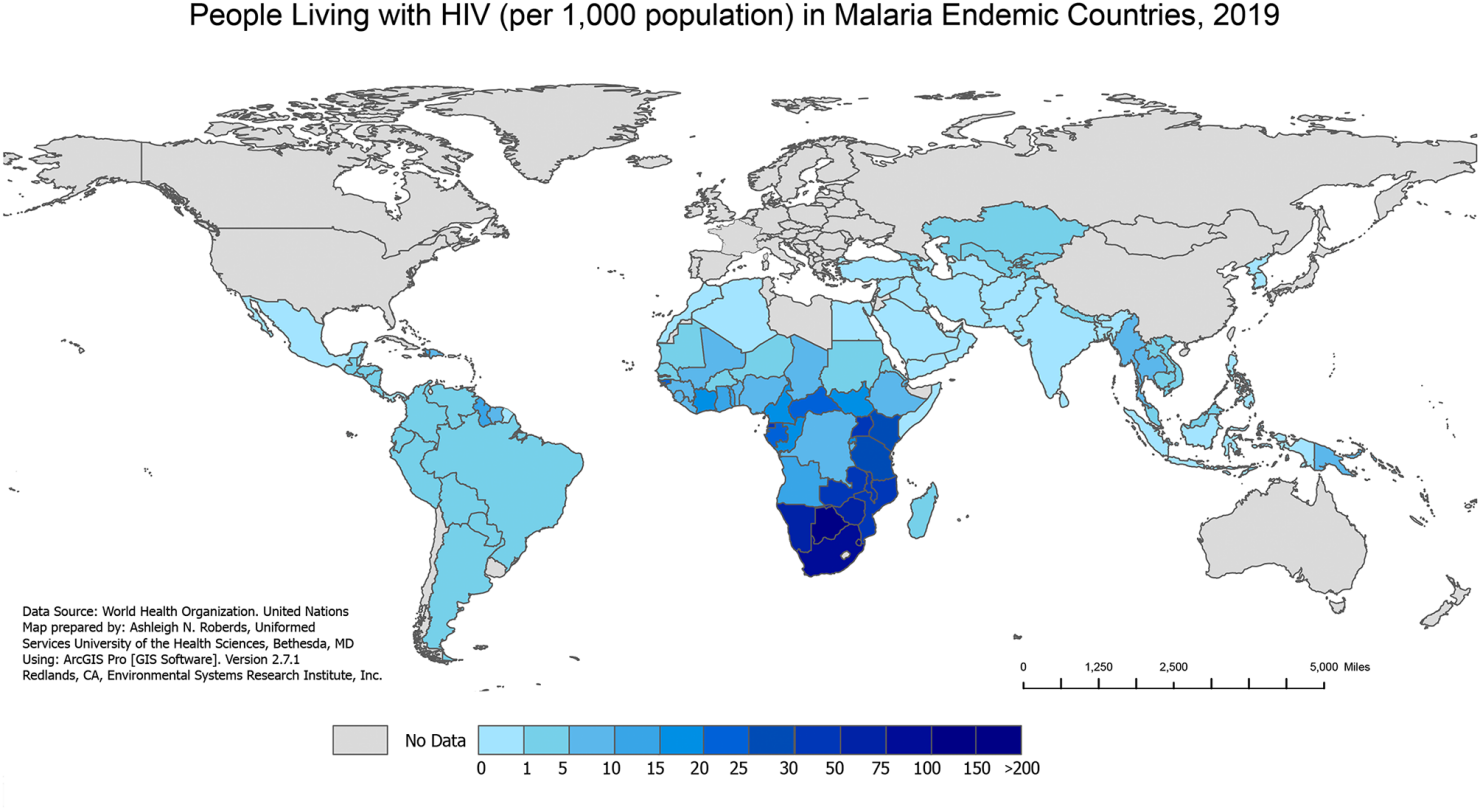
People Living with HIV in Malaria Endemic Countries, 2019. The number of people living with HIV (per 1,000 population) in 2019 in malaria endemic countries from the WHO 2020 World Malaria Report. The 2019 HIV data are extracted from the Joint United Nations Programme on HIV/AIDS (UNAIDS) AIDSinfo data sheet. The 2019 country populations are extracted from the United Nations 2019 World Population Prospects.

Over the past several decades, malaria/HIV-1 co-infection has become a significant global public health problem in co-endemic areas of the world. The geographical overlap between both diseases (**Figures 1** and **2**) combined with shared social determinants of health may explain the prevalence of co-infection, especially in sub-Saharan Africa (SSA). The distribution of diseases throughout SSA varies by country and localities, and can be explained by differing geographical, environmental, and population behaviors. However, some severely affected countries in SSA have an HIV-1 prevalence in adults above 10%, with more than 90% of the population exposed to malaria (World Health Organization, 2004). Numerous models have tried to predict the impact that malaria/HIV-1 co-infection has on incidence and mortality of each disease (Korenromp et al., 2005) but the biological dynamics within and between the two diseases are highly complex.

Data across epidemiologic populations of interest have consistently shown HIV-1-mediated immune deficiency is associated with higher prevalence of clinical malaria and increased parasite density (Hewitt et al., 2006; Flateau et al., 2011). A smaller number of studies have shown an impact of malaria co-infection enabling the progression of HIV-1, as measured by increased viral load in co-infected patients compared to those who were not co-infected (Kublin et al., 2005; Kiyingi et al., 2010). Critical factors associated with parasite transmission such as host anemia and gametocyte density are likely altered when the host is infected with HIV-1 as well. This review focuses on parasite and host factors that influence malaria parasite transmission and highlights the efforts being made to elucidate the impact that HIV-1 may have on malaria transmission potential. Consequently, this review acknowledges a substantial gap in research and understanding as the world continues to combat these two major global health priorities.

## Background

### Overview of Malaria Parasite Transmission

Significant advances have been made in reducing the burden of malaria through classic vector control, case detection and treatment strategies; however, in an era focused on malaria eradication, more attention is being diverted to reduction and prevention of transmission. For all *Plasmodium* species, transmission occurs when the female *Anopheles* mosquito vector ingests both male and female gametocytes during a blood meal from an infected host. While the physical act of transmission seems simple, the molecular dynamics surrounding successful transmission are complex. Specific developmental and metabolic events must occur within the mammalian host before mosquito infection can occur. Similarly, intricate physiological events within the mosquito vector are necessary for onward transmission to a new mammalian host.

Within the mammalian host, the parasite undergoes gametocytogenesis in which the parasite differentiates between asexual (associated with symptoms) and sexual (associated with transmission) replication. Although numerous studies have expanded upon foundational work by Bruce et al. (1990) to explain the mechanisms of sexual commitment and gametocytogenesis, the entirety of the process is not completely understood. Josling et al. (2018) provides a recent review of the significant progress in identification of molecular mechanisms surrounding parasite sexual differentiation. Foundational and novel studies have identified various factors that correlate with an increase in gametocytogenesis. These include, but are not limited to, host immunity (Smalley et al., 1981; Graves et al., 1988; Motard et al., 1995; Buckling et al., 1999), regulation of the key parasite genes GDV1 and AP2-G (Day et al., 1993; Eksi et al., 2012; Brancucci et al., 2014; Coleman et al., 2014; Kafsack et al., 2014; Filarsky et al., 2018; Josling et al., 2020), host lipid and biomolecule biosynthesis and metabolism (Gulati et al., 2015; Brancucci et al., 2017; Tanaka et al., 2019; Tewari et al., 2020), fluctuations in host hormone production (Trager and Gill, 1992; Lingnau et al., 1993), and chemotherapy (Puta and Manyando, 1997; Robert et al., 2000; Tjitra et al., 2002; Hamel et al., 2005; Elbasit et al., 2006; Schneider et al., 2006). Each of these triggers may be altered in the context of a co-morbidity such as HIV-1.

Once sexual commitment occurs, individual developing schizonts produce either all female or all male gametocytes (Silvestrini et al., 2000; Smith et al., 2000). The quantitative balance between male and female gametocytes is hypothesized to contribute to the success of malaria parasite transmission (Mitri et al., 2009). As reviewed by Henry et al. (2019) several host and parasite factors can alter the gametocyte sex ratio and ultimately impact transmission potential. Host factors include anemia and erythropoiesis (Paul et al., 2000a; Reece et al., 2005; Sowunmi et al., 2008; Sowunmi et al., 2009; Bousema et al., 2011), changing immunity (Smalley and Brown, 1981; Reece et al., 2008; Reece et al., 2009; Ramiro et al., 2011) and alterations in lipid profiles, each of which are also factors associated with HIV-1 as discussed below. Parasite factors include parasite density and gametocyte density (Robert et al., 2003; Talman et al., 2004; Schneider et al., 2007; Mitri et al., 2009), as well as parasite diversity and competition (Taylor et al., 1997; Reece et al., 2008; Babiker et al., 2008; Bousema et al., 2008; Sowunmi et al., 2009; Vardo-Zalik and Schall, 2009).

Very few studies have extensively and/or thoroughly included gametocyte and mosquito infectivity data. Successful transmission occurs only after complete gametocyte maturation in the host is followed by complete parasite fertilization and development in the mosquito vector. Results from Muirhead-Thompson (Muirhead-Thomson, 1954) cautioned investigators that concluding infectivity/transmissibility solely by gametocyte count may be misleading, and encouraged combining gametocyte data with oocyst or sporozoite enumeration data from mosquito feeding. The presence of one or more oocysts in the midgut is sufficient for transmission success.

### Other Factors That Influence Transmission

While numerous factors have the potential to influence malaria parasite transmission, immunogenetics and fluctuations in immune responses are widely studied topics. It is well described that in malaria-endemic regions, including SSA, residents are likely to have innate genetic adaptations and acquired resistance to malaria (Kwiatkowski, 2005). In 2010, Lawaly et al. reviewed the role that many of these human genetic factors play in transmission and highlighted genetic associations with asymptomatic gametocyte carriers (Lawaly et al., 2010).

A study in an endemic region in West Africa showed the ability to clear chloroquine-resistant parasites was most strongly associated with age, hematocrit, and ethnic background (Djimde et al., 2003; Kamya et al., 2006). Patients over four years of age had a dramatic increase in the ability to clear parasites, suggesting repeated exposure to malaria is a dominant factor in acquired immunity (Djimde et al., 2003). Age as a surrogate for acquired protective immunity against asexual parasites densities is supported by numerous other studies (Rogerson et al., 2010). A cross-sectional study in Burkina Faso revealed that the prevalence of gametocyte-positive carriers decreased with age. However, this study also revealed that the median proportion of gametocytes relative to asexual parasites increased with age (Ouedraogo et al., 2010). These findings suggest that adults, especially those with acquired immunity and asymptomatic infections, may be important infectious reservoirs.

In many of the regions where malaria is endemic, co-infections with other neglected tropical diseases (NTDs) and non-NTDs such as HIV-1 and tuberculosis are common. Due to the intense pressure on the host immune system to control malaria parasite infection, any immune disruption from a competing infection could adversely affect clinical outcomes and gametocyte production/clearance. Amongst NTDs, polyparasitism with *Plasmodium* spp. provides a minor protective role (Nacher et al., 2000) but more often increases severe complications as well as prevalence, density, and infectivity of asexual parasites and gametocytes (Nacher et al., 2001; Noland et al., 2007; Nacher, 2011; Ateba-Ngoa et al., 2016; Moriyasu et al., 2018). Additionally, while many studies seek to explain how malaria impacts HIV-1 disease progression and HIV-1 transmission [reviewed in (Flateau et al., 2011)], fewer studies have been designed to evaluate the converse.

## HIV Impacts on Malaria Parasite Transmission

**Figure 3:**
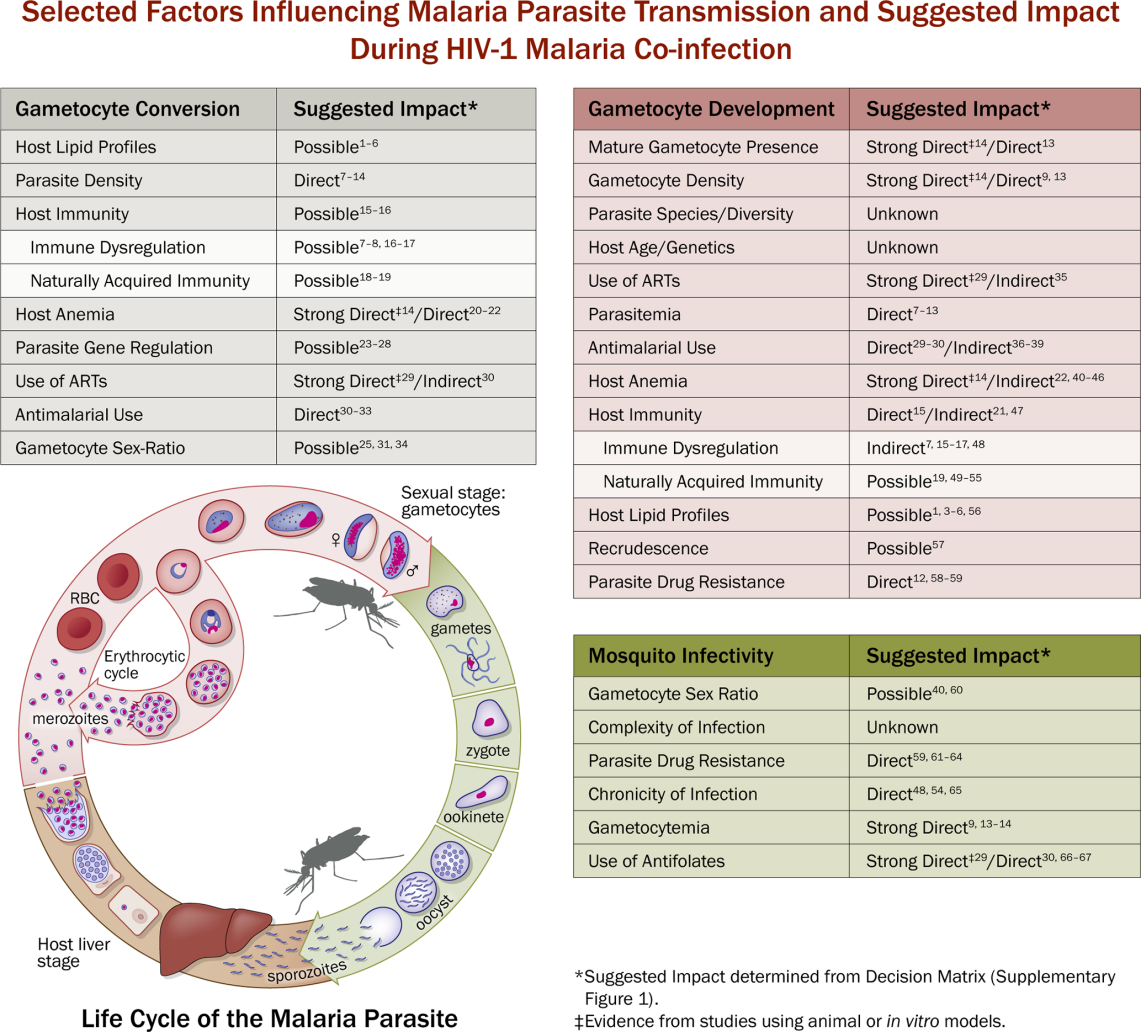
Malaria Life Cycle and Selected Factors Influencing Malaria Parasite Transmission and Suggested Impact During HIV-1 Malaria Co-infection. Example of selected factors that are known to be associated with malaria parasite transmission and the suggested impact (determined using Decision Matrix, **Supplementary Figure 1**.) with HIV-1 and/or malaria/HIV-1 co-infection. Suggestive impact is used due to the lack of available statistical evidence and the difficulty in ascertaining the biological relationship between malaria/HIV-1 co-infection and malaria parasite transmission success. Strong Direct Impact means that there is evidence, which includes mosquito transmission data, to suggest that HIV-1 malaria co-infection directly alters gametocyte conversion (GC), gametocyte development (GD), or mosquito infectivity (MI). Direct Impact means there is evidence, sans mosquito data, to suggest that HIV-1 malaria co-infection directly alters GC, GD, or MI. Indirect impact means there is evidence to suggest that HIV-1 or HIV-1 malaria co-infection indirectly alters factors that influence GC, GD, or MI. Possible Impact means there is evidence to suggest that HIV-1 or HIV-1 malaria co-infection may alter factors associated with GC, GD, or MI. Unknown Impact means there is currently not enough evidence to suggest that HIV-1 or HIV-1 malaria co-infection directly or indirectly alter GC, GD, MI, or the factors associated with GC, GD, or MI. The left table (gray, “Gametocyte Conversion”) represents selected transmission factors that pertain to the gametocytogenesis and gametocyte commitment. The center table (red, “Gametocyte Development”) represents selected transmission factors that pertain to the sexual stage development. The right table (green, “Mosquito Infectivity”) represents selected factors that may influence infectivity to mosquitoes and subsequent mosquito transmission success. In the middle is a representation of the life cycle of *Plasmodium falciparum* (adapted from NIAID/NIH, 2016, https://www.niaid.nih.gov/diseases-conditions/malaria-parasite) representing the exo-erythrocytic (liver) cycle, erythrocytic cycle (bloodstream), and sexual stage (gametocyte) development in the host, and the sporogonic cycle in the mosquito. ^1^Brancucci et al., 2017; ^2^Tanaka et al., 2019; ^3^Orikiiriza et al., 2017; ^4^Drobnik et al., 2003; ^5^Belury et al., 2003; ^6^Bowman et al., 2019; ^7^Whitworth et al., 2000; ^8^Patnaik et al., 2005; ^9^Abu-Raddad et al., 2006; ^10^Laufer et al., 2006; ^11^Onyenekwe et al., 2007; ^12^Van Geertruyden et al., 2008; ^13^Stiffler et al., 2020; ^14^Trott et al., 2011; ^15^Koehler et al., 2009; ^16^Okonkwo et al., 2016; ^17^Mbale et al., 2016; ^18^Graves et al., 1988; ^19^Nassir et al., 2005; ^20^Lingnau et al., 1993; ^21^Bousema et al., 2011; ^22^Belperio and Rhew, 2004; ^23^Eksi et al., 2012; ^24^Brancucci et al., 2014; ^25^Josling et al., 2020; ^26^Coleman et al., 2014; ^27^Kafsack et al., 2014; ^28^Filarsky et al., 2018; ^29^Hobbs et al., 2013; ^30^Azevedo et al., 2019; ^31^Buckling et al., 1999; ^32^Elbasit et al., 2006; ^33^Ittiravivongs et al., 1984; ^34^Josling et al., 2018; ^35^Sandison et al., 2011; ^36^Puta and Manyando, 1997; ^37^Schneider et al., 2006; ^38^Barnes et al., 2008; ^39^Bouyou Akotet et al., 2018; ^40^Sowunmi et al., 2008; ^41^Quintero et al., 2011; ^42^Nacher et al., 2002; ^43^Tay et al., 2015; ^44^Sanyaolu et al., 2013; ^45^Okechukwu et al., 2018; ^46^Van Geertruyden et al., 2009; ^47^Stone et al., 2018; ^48^Kamya et al., 2006; ^49^Schneider et al., 2007; ^50^Babiker et al., 2008; ^51^Djimde et al., 2003; ^52^Rogerson et al., 2010; ^53^Tadesse et al., 2018; ^54^Wargo et al., 2007; ^55^Tukwasibwe et al., 2014; ^56^Gulati et al., 2015; ^57^Birku et al., 2002; ^58^Oesterholt et al., 2009; ^59^Torrevillas et al., 2020; ^60^Paul et al., 2000b; ^61^Robert et al., 2000; ^62^Hogh et al., 1998; ^63^Beavogui et al., 2010; ^64^Kone et al., 2010; ^65^Kakuru et al., 2013; ^66^Kamya et al., 2007; ^67^Mermin et al., 2006.

The impacts/effects of HIV-1 infection on malaria parasite transmission are often defined as direct or indirect, with the former being very difficult to identify due to the complexities of both diseases (**Figure 3**). An increase in parasitemia or parasite biomass is the most cited association with malaria/HIV-1 co-infection, but the direct relation to gametocytemia or transmission potential is often suggestive or speculative (Whitworth et al., 2000; Patnaik et al., 2005; Abu-Raddad et al., 2006; Laufer et al., 2006; Onyenekwe et al., 2007; Van Geertruyden et al., 2008). There have been various *in vivo* and epidemiological studies that seek to further explain the impact of HIV-1 on gametocytogenesis and gametocytemia, but most studies detail only indirect effects. Interesting data from a study of asymptomatic malaria among individuals who were either HIV-1 negative or positive suggests that HIV-1 positive individuals have a greater risk of carrying gametocytes than HIV-1 negative individuals (Stiffler et al., 2020). This study is the first of its kind designed to evaluate the epidemiological impact of HIV-1 co-infection on the prevalence of asymptomatic gametocytemia in the field.

Although there have been advances in humanized mouse models and non-human primate models for HIV-1 and malaria co-infection studies (reviewed in (Hatziioannou and Evans, 2012; Vaughan et al., 2012; Beignon et al., 2014)), there has been little research conducted in the laboratory to directly investigate co-infection and parasite transmission. In 2009, Koehler et al. described a non-human primate model for co-infection, but the study was not intended to provide any direct evidence related to malaria parasite transmission. In 2011, Trott et al. designed a study to evaluate effects of simian immunodeficiency virus (SIV) co-infection on malaria parasite transmission in a non-human primate model (Trott et al., 2011). The study modeled an underlying immunodeficiency virus infection prior to a subsequent malaria parasite infection by inoculating rhesus macaques with SIV followed by inoculation of *Plasmodium fragile*, approximately 28 days later. Results from the study showed that co-infection was associated with a significantly increased risk of malaria parasite transmission and that SIV had a direct enhancing effect on parasitemia, gametocytemia and mosquito infectivity (Trott et al., 2011). The *P. fragile* model resembles human *P. falciparum* infections especially in the context of severe disease and parasite sequestration (Craig et al., 2012). Additional factors may have contributed to an indirect effect on increased transmission, such as immune regulation and reduced hematocrit levels. Additionally, it is difficult to extrapolate this acutely infected animal model with the more typical chronically infected situation in humans in malaria endemic areas.

### Host Factors Affected by HIV That Could Perpetuate Malaria Parasite Transmission

To date, no clinical studies have looked directly at parasite transmission in HIV-1-infected patients. However, patients with progressive HIV-1 disease are known to have a number of disease-related sequelae, including immune deficiency, anemia and alterations of lipid profiles. Further, HIV-1-positive patients are commonly prescribed antiretroviral and antifolate medications. Numerous physiologic changes that occur during HIV-1 infection independently are known to affect malaria parasite transmission, some positively and some negatively. Here we present data from a variety of studies suggesting that HIV-1-infected patients are at increased risk of being reservoirs for malaria parasite transmission.

#### Immune Status

A review by Troye-Blomberg (1994) highlighted the importance of both cell-mediated and humoral immunity to asexual blood stages of malaria. A later study by Goodier et al. found that gametocytes activate a CD4+ T-cell response (Goodier and Targett, 1997). More recent reviews have confirmed these early findings, affirming that T-cells and cytokines suppress gametocyte burden and decrease infectivity to mosquito vectors (Kengne-Ouafo et al., 2019). When a host mounts a humoral immune response against gametocyte-specific antigens, the antibodies produced are taken up during a mosquito blood meal, and these have been shown to inhibit fertilization and/or parasite development (anti-gamete immunity) with a concomitant decrease in mosquito infection (Bousema and Drakeley, 2011; Stone et al., 2018; Kengne-Ouafo et al., 2019).

These immunologic findings have been supported by field data on malaria parasite transmission. A study in Gambia found that the absence of high fever and of high parasite densities were independent risk factors for gametocytemia (von Seidlein et al., 2001). The authors hypothesized that fever and high parasite densities in those with a history of infection represented acute infection with an active immune response. Lack of an acute infection could prevent gametocyte exposure to important damaging elements of the immune system, such as inflammatory cytokines (von Seidlein et al., 2001). A more recent study in Ghana identified relatively lower prevalence of gametocytemia in patients with fever (Dinko et al., 2018). *In vitro* findings demonstrated that increased levels of cytokines (including TNF-α and IFN-γ) correlated with a lack of gametocyte infection of the mosquito vector (Naotunne et al., 1993). These data suggested that patients with clinical malaria may be less likely to develop gametocytemia, or to serve as good reservoirs for transmission.

Effects of immune deficiency were studied by Koehler et al. in a rhesus macaque model of SIV-*P. cynomolgi* co-infection (Koehler et al., 2009). They found significant effects of co-infection on the immune response to malaria parasite infection, including a more rapid depletion of CD4+ T-cells, a failure to generate an appropriate CD4+ T-cell response to parasitemia, and decreased proliferative B-cell response (anti-parasite IgG) (Koehler et al., 2009). This study did not directly examine effects on transmission to mosquitoes, but the data provided convincing evidence that co-infection impacts the production of key immunological factors that control gametocytemia.

The inability of an HIV-infected host to mount a robust immune response against malaria infection is expected based on the depletion of CD4+ T cells during HIV-1 infection. Several clinical studies suggest a relationship between the severity of immune deficiency, specifically decreased CD4+ T-cell counts and repressed cytokine synthesis, and worsening malarial disease. A long-term study in Uganda followed nearly 500 HIV-positive participants and controls over an eight-year period to determine the frequency of clinical malaria. The immune deficiencies of the HIV-1-positive patients were categorized according to the WHO hierarchical classification of CD4+ T-cell count. Results showed a positive association between increasing immunosuppression and increasing parasitemia. Further, the odds of having clinical malaria were six times higher in patients with CD4+ T-cell counts less than 200/μl as compared to those with counts higher than 500/μl (Whitworth et al., 2000). A second large cohort study explored the effects of HIV-1 co-infection on the outcomes of cerebral malaria in children, finding that children without HIV-1 had substantially increased levels of TNF-α and ICAM-1 during their clinical malaria episodes. Despite the varied inflammatory responses, time to parasite clearance was similar in HIV-1-infected and -uninfected groups (Mbale et al., 2016). A third study by Laufer et al. investigated the impact of HIV-1-associated immunosuppression on malaria in patients in Malawi (Laufer et al., 2006). The study showed that parasite density in symptomatic clinical infection was inversely related to CD4+ T-cell count, a relationship that was not seen in patients with asymptomatic infections. In contrast, a cross-sectional study in Nigeria observed that the prevalence of asymptomatic malaria parasitemia in children under five was significantly higher in HIV-1 co-infected children, was highest among HIV-1 co-infected children who were severely immunosuppressed and was significantly associated with declining CD4+ T-cell counts (Okonkwo et al., 2016). Each of these studies failed to measure gametocytemia specifically, but given our current understanding of the importance of T-cells and cytokines in immunity to asexual parasites, it is possible that a similar association exists between decreasing CD4+ T-cell counts and blunted cytokine responses in HIV-infected patients and increasing gametocytemia. However, special consideration should be given to asymptomatic malaria carriers as they represent a unique and challenging demographic due to partial protective immunity acquired with age and exposure in endemic areas.

#### Hematocrit Levels

Malaria is known to cause anemia by multiple mechanisms, including destruction of infected erythrocytes, antibody-mediated bystander erythrocyte lysis, and impaired erythropoiesis (Quintero et al., 2011). A large study by Nacher et al. examined host factors influencing *P. falciparum* gametocyte carriage and found that hemoglobin concentrations (as a measure for anemia) were negatively correlated with peak gametocyte counts and gametocyte carriage duration (Nacher et al., 2002). This association between gametocytemia and anemia has been reported in other studies (Price et al., 1999; Stepniewska et al., 2008; Dinko et al., 2018), suggesting that anemia, and thus perhaps tissue hypoxia, stimulates *P. falciparum* gametocytogenesis. A large Nigerian study of 1125 children attempted to discern the mechanism by which anemia influences gametocyte development and found the proportion of male to female gametocytes in anemic children was nearly two-fold higher than in non-anemic children. The authors suggested that anemia may prolong the half-life of male gametocytes and potentially their survival in circulation (Sowunmi et al., 2008).

Possible mechanisms by which anemia triggers gametocyte development are well reviewed by Bousema and Drakeley (2011). Anemia in a host is associated with increases in both relative and absolute levels of the immature red blood cells called reticulocytes. Reticulocytes have high RNA content and increased hemoglobin synthesis and are stimulated by the glycoprotein cytokine erythropoietin (EPO). EPO has been implicated as a stimulant for gametocyte formation in *P. chabaudi* and *P. vinckei* (Reece et al., 2005) and is thought to influence gametocyte sex allocation (Paul et al., 2000b). These conditions in an anemic host are postulated to be ideal for gametocyte development (Bousema and Drakeley, 2011), but cause-and-effect between reticulocytemia and gametocyte levels has not been elucidated.

Anemia is also a common clinical finding in HIV-1 infection, with prevalence up to 95% depending on patient factors such as stage of HIV-1 disease, sex, age, and pregnancy status (Belperio and Rhew, 2004). Because anemia is not unique to either disease, determining the effect of HIV-1-related anemia on malaria parasite transmission is challenging. Trott et al. examined the effects of immunodeficiency virus infection on malaria parasite transmission using SIV/*P. fragile* co-infection in rhesus macaques. These authors found an increase in the percentage of gametocytes during the initial drop in hematocrit in both parasite- infected and co-infected animals (Trott et al., 2011). The mechanism by which anemia in these animals induced gametocytemia is unclear, but these data suggested that immunodeficiency virus-induced anemia could be associated with higher risk of malaria parasite transmission.

Data from epidemiologic field studies examining this association are difficult to interpret, as most studies fail to associate the prevalence of anemia with increased risk of parasite transmission beyond measures of gametocytemia. Tay et al. studied 400 HIV-1 seropositive patients with and without malaria co-infection and found an overall anemia prevalence of 67%, with a prevalence of nearly 94% in HIV-malaria co-infected patients (Tay et al., 2015). A second study found median hemoglobin levels were lower in HIV-1 positive patients with positive malaria blood smears than HIV-1 positive patients without malaria (Sanyaolu et al., 2013). In pregnant women in Nigeria, low hemoglobin levels were correlated with malaria/HIV-1 co-infection, but not with decreasing CD4+ T-cell counts (Okechukwu et al., 2018). Only one study in Zambia found HIV-1-infection, not CD4+ T-cell count, to be an independent risk factor for a longer duration of anemia in co-infected patients (Van Geertruyden et al., 2009). These studies clearly demonstrate that anemia is prevalent in co-infected patients but did not provide data to support that anemia in HIV-1-infected patients is a significant risk factor for increased malaria parasite transmission specifically. Additionally, it is difficult to discern if the primary etiology of anemia in these patients is HIV-1 infection or malaria, given that anemia is common in both diseases as well as other NTDs prevalent in these settings. More studies in co-infected patients are needed to determine if data from co-infected animal models are reproducible in humans.

#### Lipid Profiles

A recent *in vitro* study by Brancucci et al. (2017) concluded that levels of host-derived lipids, specifically lysophosphatidylcholine (LysoPC), could act as an environmental stimulus for *P. falciparum* gametocyte differentiation. Specifically, *Plasmodium* parasites use LysoPC for phosphatidylcholine (PC) biosynthesis and the resultant depletion of LysoPC leads to dramatic induction of gametocytogenesis (Brancucci et al., 2017). This association between the host lipid profile and gametocytogenesis is clinically relevant given that systemic LysoPC levels are altered throughout the course of a malaria infection. The most common fluctuations are often associated with the host immune response to disease progression and parasitemia (Drobnik et al., 2003; Orikiiriza et al., 2017). A study on lipid profiles in HIV-1 infection demonstrated increased levels of LysoPC in HIV-1-positive patients pre- and post-antiretroviral therapy (ART) compared to HIV-1-negative patients (Belury et al., 2017). A recent study by Bowman et al. showed that the concentration and fatty acid composition of LysoPC differed between HIV-1-positive and HIV-1-negative patients. Although there were no significant differences between serum concentrations of total LysoPC, the species of LysoPCs showed differential enrichment. In particular, LysoPCs containing saturated fatty acids (SaFAs) were enriched, while LysoPCs containing polyunsaturated fatty acids (PUFAs) were reduced in HIV-1-positive patients. SaFA-enriched LysoPCs were also associated with immune activation in HIV-1-positive patients, consistent with elevated serum levels of interleukin-6 (IL-6) and markers of monocyte activation (Bowman et al., 2019). Understanding the clinical relevance of these observations is in the early stages, but alterations in lipid profiles of HIV-1-infected patients could influence gametocyte production and transmission to mosquito vectors.

### Impact of Therapeutic Agents

#### Antifolates

In populations most affected by malaria and HIV-1 co-infection, use of ART and antifolate prophylaxis are important to consider when discussing host factors that impact malaria parasite transmission. Excellent laboratory studies suggested direct effects of various antiretroviral agents, particularly cysteine protease inhibitors, on gametocytogenesis and malaria parasite transmission, which have been recently reviewed (Azevedo et al., 2019). Antifolate drugs, however, have been used both as primary antimalarial agents (e.g., Sulfadoxine-Pyrimethamine or SP) and as antibiotics adjuncts to ART (e.g., trimethoprim-sulfamethoxazole or TMP-SMX) for the prevention of opportunistic infections in HIV-positive patients.

Two early studies in the 1970’s failed to show an effect of TMP-SMX on gametocytemia, though both studies were limited by a small sample of patients (Wilkinson et al., 1973; Hansford and Hoyland, 1982). Research interest has grown dramatically over the last several decades and has revealed that SP monotherapy may increase gametocytemia in the early stages of treatment (Puta and Manyando, 1997; Schneider et al., 2006; Barnes et al., 2008), but long-term TMP-SMX therapy could decrease parasite burden and transmission (Mermin et al., 2006; Kamya et al., 2007; Hobbs et al., 2012; Hobbs et al., 2013).

Early studies in the 1980’s began to associate growing resistance of *P. falciparum* to the use of SP (Ponnampalam, 1982; Kupferschmidt et al., 1988), though these data conflicted with a small study which failed to find a stimulating effect of SP on gametocytogenesis (Ittiravivongs et al., 1984). In 1997, a study by Puta and Manyando (1997) measured gametocytemia in patients treated with SP or chloroquine and found a significant difference between the two treatment groups. In 2006, Schneider et al. (2006) convincingly showed the risk of gametocyte carriage and density in Kenyan children with falciparum malaria was significantly higher in patients treated with SP monotherapy compared to those treated with combination SP and artesunate (AS) therapy. Further, gametocyte prevalence and density decreased over time in patients with SP+AS therapy but not in SP-treated children.

These studies suggested increasing levels of *P. falciparum* resistance to SP, that was confirmed by Barnes et al. (2008) in 2008. These authors conducted three therapeutic efficacy studies in *P. falciparum*-infected patients treated with SP over the course of five years. Notable findings included a significant increase in post-treatment gametocytemia with most significant increases in gametocyte positivity rates between days 14 and 21 of treatment. Over the course of the study, the mean maximum gametocyte density in patients increased seven-fold from 2000 to 2002, and this was attributed to a rapidly increasing frequency of Pf*dhfr/dhps* mutations, encoding SP resistance. Oesterhalt et al. (Oesterholt et al., 2009) showed a relationship between submicroscopic gametocytemia and the presence of Pf*dhfr* mutations in areas of East Africa that previously reported adequate response to SP treatment. These growing resistance patterns lead to the institution of artemisinin-based therapy as the primary treatment in 2006 (Gregson and Plowe, 2005; Amin et al., 2007), though SP is still prescribed for intermittent preventive treatment in pregnancy (WHO) and as prophylaxis for HIV-1-associated opportunistic infections in malaria endemic areas.

While SP monotherapy has been associated with increased risk of gametocytemia, subsequent infectivity to mosquitoes surrounding these observations has been inconclusive (Hogh et al., 1998; Buckling et al., 1999; Robert et al., 2000; Schneider et al., 2006; Beavogui et al., 2010; Kone et al., 2010). In particular, TMP-SMX prophylaxis has been shown to reduce the risk of parasite transmission in malaria/HIV-1 co-infection. Hobbs et al. in 2012 (Hobbs et al., 2012) showed that TMP-SMX treatment of mice infected with *Plasmodium berghei* or *Plasmodium yoelii* significantly reduced liver stage parasite burden and peripheral parasitemia. These data were foundational for a second study by Hobbs et al. in 2013 (Hobbs et al., 2013) focusing on the effect of ART and antifolates on two key aspects of parasite transmission - gametocyte burden and mosquito infectivity. In this study, strains of *P. falciparum* were exposed to TMP-SMX at concentrations equivalent to those in HIV-positive patients receiving prophylactic treatment. Unmetabolized TMP-SMX did not significantly reduce gametocyte viability or inhibit gametocyte exflagellation *in vitro*, but did reduce oocyst infection in mosquitoes. These findings suggested the possibility that TMP-SMX might impact mosquito infectivity directly, thus, potentially reducing transmission.

In HIV-1-malaria co-infected patients, numerous clinical studies have suggested that antifolate treatment has an inhibitory effect on parasite transmission. A large cohort study of 300 HIV-1-infected children in Uganda showed that TMP/SMX treatment and provision of insecticide-treated bed nets reduced malaria incidence by 97%, despite geographically high levels of antifolate resistance (Kamya et al., 2007). Similar effects were seen in a cohort study of over 1000 Ugandan adults, showing that TMP-SMX treatment led to an incidence of 9 clinical episodes of malaria per 100 person years compared to a baseline incidence of 50.8 clinical episodes per 100 person years (Mermin et al., 2006). TMP-SMX prophylaxis also showed promising effects in HIV-1 exposed infants, reducing malaria incidence by 39% relative to HIV-1-exposed infants who did not continue therapy (Sandison et al., 2011).

A particularly intriguing study by Mermin et al. in 2005 (Mermin et al., 2005) examined a large sample of HIV-infected patients and their HIV-1-negative family members and found that TMP-SMX prophylaxis in HIV-1-positive patients significantly reduced malaria disease burden in HIV-1-negative family members. While reductions in malaria-associated morbidity, infection rates, and hospitalization are unquestionably multifactorial, it is possible that antifolates are directly anti-parasitic. Further, although the study did not report malaria incidence for HIV-1-positive individuals, it could reasonably be surmised that malaria was prevalent in the HIV-1-population given the known increased risk of malaria in HIV-1-positive patients. Future studies similar in design but targeted at HIV-1-malaria co-infected patients and including mosquito infectivity studies, could further support a direct impact of antifolate therapy on malaria parasite transmission.

#### Malaria Treatment Failure

Malaria treatment failure, as defined by clinical failure, parasitological failure, or recrudescence, is a significant problem for efforts to mitigate clinical infection and control parasite burden. Though this issue is multi-faceted, encompassing vector biology, innate host immunity, drug resistance, and environmental considerations, this scope of challenges now extends to the impact of HIV-1 on parasite clearance, including clearance remediated by antimalarial treatment. Birku et al. (2002) investigated the effect of artemisinin on clearance of *P. falciparum* in patients with and without HIV-1 co-infection. Their data showed increased time for parasite clearance and inability to entirely clear parasitemia within a designated treatment period. Further, mean parasite density was 12-fold higher in HIV-1 seropositive patients than in seronegative controls. A second large retrospective study of 1965 patients in Uganda revealed similar findings, showing patients with HIV-1 co-infection had a greater than 3-fold increased risk of antimalarial treatment failure. Molecular analysis of these treatment failures indicated these failures were due to new infections rather than recrudescence (Kamya et al., 2006). In a large cohort study of Ugandan children, Kakuru et al. (2013) studied how different HIV-1 treatments and prophylaxis affected gametocytemia in children and found that dihydroartemisinin-piperaquine (DP) and TMP-SMX treatment were associated with an increased risk both of any gametocytemia and of failed gametocyte clearance during malaria follow-up. Additional studies have shown that many of the antimalarials recommended globally have little to no effect on gametocytes and may allow gametocytes to persist for more than 1 month after successful clearance of asexual parasites (Bousema et al., 2006; Bousema et al., 2010). Beyond the clinical challenges of treatment failure, an inability to control parasitemia and gametocytemia may make HIV-1-infected patients an enhanced reservoir for transmission.

### Population of Special Interest: Asymptomatic Individuals

The presence of gametocytes in a human host has not been associated with any distinct clinical findings. Gametocytes can be detected in acutely symptomatic malaria patients as well as asymptomatic carriers, especially in endemic areas where multiple infections are common and clinical immunity develops during childhood. Asymptomatic infections pose a unique threat to malaria control as they represent a large reservoir of hosts capable of unknowingly harboring and transmitting parasites. Recent evidence (based on hypotheses from the early 1900s) indicates that many asymptomatic individuals harbor gametocytes at such low densities that they are not often detected by microscopy [reviewed in (Nassir et al., 2005; Babiker et al., 2008)]. Associations between asymptomatic infections with low parasite/gametocyte density and mosquito infectivity vary significantly in relation to parasite genetic diversity (complexity of infection), drug resistance, and host immunity/chronicity of infection (Wargo et al., 2007; Bousema and Drakeley, 2011; Tukwasibwe et al., 2014).

At this time, the prevalence of HIV-1 co-infected asymptomatics has not been fully described due to the difficulty in ascertaining asymptomatic malaria patients. Two large studies of adults in Africa showed conflicting results in overall prevalence of asymptomatic parasitemia, with HIV-1 positive patients showing significantly lower prevalence of asymptomatic parasitemia than HIV-1 negative controls. HIV-1 positive patients not using TMP-SMX prophylaxis had an increased risk of parasitemia, suggesting that antifolates reduce parasitemia (Bouyou Akotet et al., 2018; Kamau et al., 2020). These findings were proposed to result from an increased tendency of HIV-1 positive patients to seek medical care and use prophylactic measures (Bouyou Akotet et al., 2018). A cross-sectional study in Kenya revealed that more than 60% of malaria asymptomatic adults seeking HIV-1 testing in the Kisumu region are positive for malaria [submitted manuscript, Kifude, C. et al., 2021], but significant differences by HIV-1 status have not emerged. Recent evidence from Kamau et al. revealed that asymptomatic malaria infections were significantly associated with abnormal hematological outcomes in people living with HIV (Kamau et al., 2020).

Contrary to parasitemia data above, asymptomatic individuals infected with HIV-1 have been shown to have a significantly higher risk of being gametocyte positive, and with a higher relative gametocyte density, compared to HIV-1-negative individuals (Stiffler et al., 2020). Further, HIV-1 co-infection is associated with significant differences in *P. falciparum dhfr* and *dhps* haplotypes in the same patient population, suggesting that HIV-1 co-infection could impact the spread of drug resistance (Torrevillas et al., 2020). These observations are concerning in the context of transmission reduction, especially considering the high prevalence of asymptomatics in varying endemnicities (Alves et al., 2005; Bousema et al., 2014; Sattabongkot et al., 2018; Tadesse et al., 2018) and areas with seasonal transmission. As reviewed by Babiker et al. (2008), asymptomatic gametocyte carriers are a likely source of seasonal epidemics as they harbor gametocytes throughout transmission-free seasons until the vector returns (Babiker et al., 1998; Nassir et al., 2005).

## Conclusions

Although the current evidence is suggestive, the bidirectional impact of co-infection on the clinical progression of both diseases suggests that HIV-1/malaria co-infection may be a catalyst for increased parasite transmission. In order to elucidate how contributing co-infection factors, define malaria parasite transmission success, combined clinical and entomological studies must be undertaken. Importantly, the nature of the entomological studies depends on the question(s) to be answered. For example, if the question is whether circulating gametocytes are intrinsically infectious, the study design may include replacing serum from a volunteer blood sample with non-immune serum to eliminate circulating host factors that could obscure intrinsic infectivity of gametocytes to exposed mosquitoes. If, on the other hand, the question is whether a volunteer is infectious based on their current clinical or treatment status, direct mosquito feeding on whole blood would be preferable to account for circulating host factors and therapeutics that could impact parasite development in the mosquito host. Given that many of the intrinsic and extrinsic host factors noted above, some of which can fluctuate on a very short timescale, can affect gametocytogenesis and gametocytemia, and that mean gametocyte circulation time in the periphery is 3.4-6.4 days (Bousema and Drakeley, 2011), matched clinical samples collected at or very close to the time of mosquito feeding are essential to directly correlate volunteer clinical status to mosquito infection success. Even with the best coordination, however, infection of mosquitoes after direct skin feeding on infected falciparum gametocyte-positive volunteers is far from uniform; the average success rate of mosquito infection from a 2012 survey of 930 feeding experiments in a variety of endemic settings was 62% (Bousema et al., 2012). Accordingly, longitudinal studies have particular value in that volunteers can be re-tested over time, perhaps multiple times per month for several months, to track both clinical and treatment profiles with mosquito infection success. Each volunteer, therefore, can provide both control (baseline) and temporal data on host factor correlates with mosquito infectivity. In this context, special consideration should be made to further understand the impact of asymptomatic gametocyte carriers, the duration and intensity of gametocyte carriage, the presence of HIV-1 co-infection and the potential effects of HIV-1 and malaria chemotherapies on experimental parasite transmission to competent mosquito vector species.

From a public health perspective, asymptomatic *Plasmodium*-infected individuals remain one of the biggest threats to malaria control and eradication programs. If HIV-1 co-infected individuals have increased prevalence, frequency, duration and/or intensity of gametocytemia, they are unwittingly and unintentionally more infectious reservoirs of parasite transmission for the rest of their community. As such, these individuals represent an ideal target for additional therapies in conjunction with ART for the protection of public health and for malaria elimination and eradication.

## Author Contributions

VAS conceived the idea for a review on the topic. AR coordinated the review, and designed and drafted all figures. AR and EF contributed to manuscript design and drafted the manuscript. All authors contributed to the article and approved the submitted version.

## Funding

This work was supported by NIH NIAID R01 AI104423 (VAS, SL).

## Disclaimer

The contents, views or opinions expressed in this publication or presentation are those of the author(s) and do not necessarily reflect the official policy or position of Uniformed Services University of the Health Sciences, the Department of Defense (DoD), or Departments of the Army, Navy, or Air Force. Mention of trade names, commercial products, or organizations does not imply endorsement by the U.S. Government.

## Conflict of Interest

The authors declare that the research was conducted in the absence of any commercial or financial relationships that could be constructed as a potential conflict of interest.
