# Supplementary figures and images for: HIV-1 Impact on Malaria Transmission: A Complex and Relevant Global Health Concern
Source: Front Cell Infect Microbiol. 2021 Apr 12;11:656938. doi: 10.3389/fcimb.2021.656938 (PMC8071860; doi:10.3389/fcimb.2021.656938)

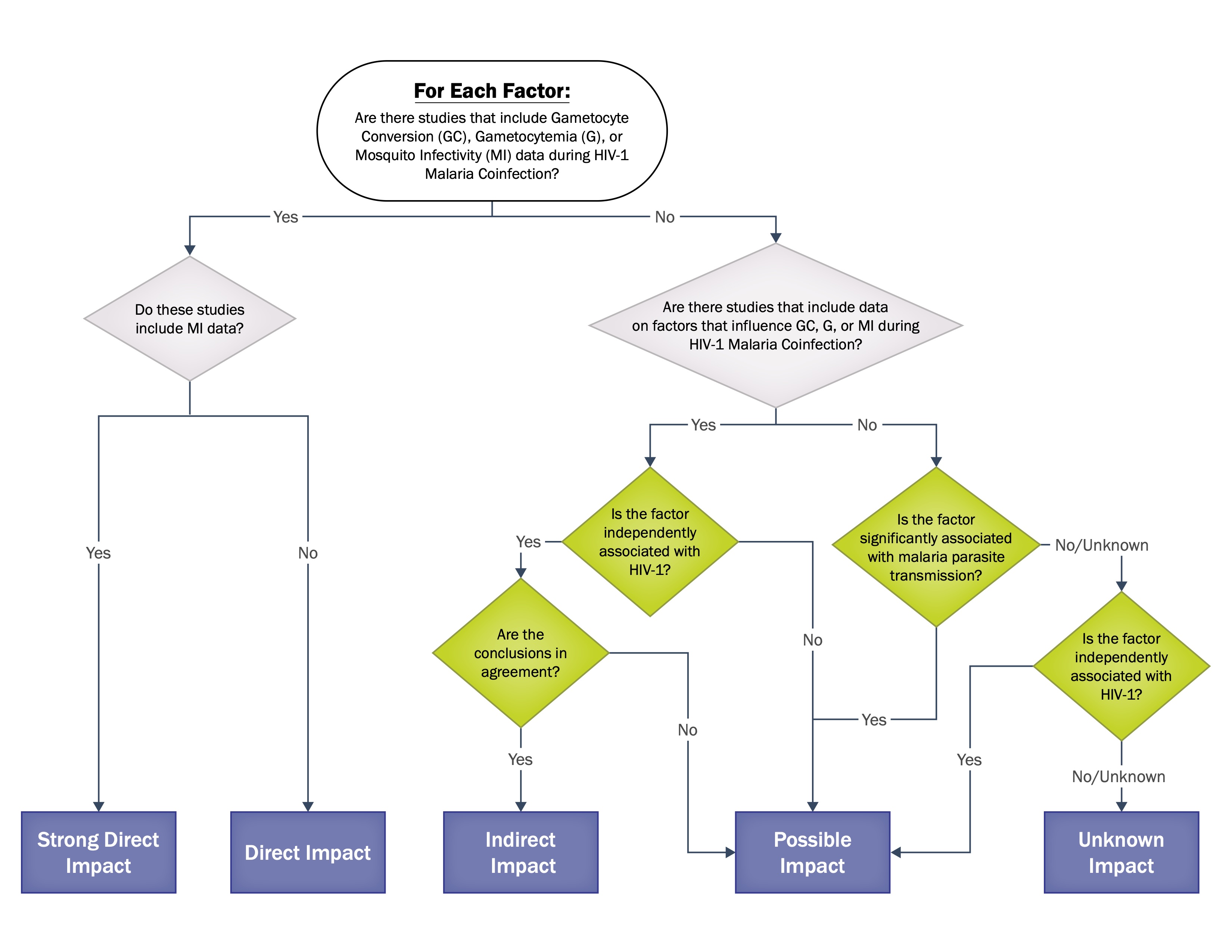

Supplement: Supplementary file 1 [file Image_1.jpeg]
